# Supplementary material for: Patterns of Intron Gain and Loss in Fungi
Source: PLoS Biol. 2004 Nov 30;2(12):e422. doi: 10.1371/journal.pbio.0020422 (PMC532390; doi:10.1371/journal.pbio.0020422)
Supplement: Table S1 — Also available at http://genes.mit.edu/NielsenEtAl/. (4.3 MB ZIP). [file pbio.0020422.st001.zip › NielsenEtAl/html/868.html]

AN5404.1.NCU06361.1.MG04197.1.FG09796.1


```
 CLUSTAL W (1.82) Multiple Sequence Alignments - Introns Inserted


Sequence 1: MG04197.1	324 aa
Sequence 2: FG09796.1	357 aa
Sequence 3: NCU06361.1	426 aa
Sequence 4: AN5404.1	318 aa
Alignment Length: 452 aa
Number Identitical Residues: 91 aa
Alignment Score (without introns) 5848


MG04197.1 	------------------------------------------------------------
NCU06361.1	MAETECGQENRRSDRVTANSDTLGLPFLWHDEIIHRIRSGGGAAPLARALRSFSCKMCAS
FG09796.1 	------------------------------------------------------------
AN5404.1  	------------------------------------------------------------
          	                                                            

MG04197.1 	--------------------------~------MADTAPAANGGSNIDALKNN1---~AA
NCU06361.1	LVDGGVLPAYPARCPIFVWWGYPAAL0QGHPNTFTMADDYSNGSTNLDALKNS~KVH1AA
FG09796.1 	--------------------------~------MADSSPVANGNSLLQTTKTN1---~AA
AN5404.1  	--------------------------~------MADNSEVR------------~---~--
          	                                 ::                         

MG04197.1 	AAYDSVTNG1PVAQNVKDQSAKTTAELNNLAASRQTPTQPAATGQPLTH~YHSFFSELLS
NCU06361.1	SAYDAVTKG1PVAQNVADQHAKTVDELSNLSASRRTPSTPAATGQPLTH~YHSFFSELLS
FG09796.1 	AAYQSVANG1PVAQNVYGHTKKASTELSNLAASRRTPANPAATGQPLTH~YHSFFSELLS
AN5404.1  	--YPDPNAS1PVAETIRSEASRTGQEFRDLKSAKVIPSTTTATGQPLTY1YHSLFYSLLS
          	  *     . ***:.: ..  ::  *: :* :::  *: .:*******: ***:* .***

MG04197.1 	~WNNPR1ASGIAYASIVTFIFAVRYLDVIRWSLKLSGYVLAT~TVAAEVVGKAILGTGFA
NCU06361.1	~WNNPR1ASAIAYLSIVAAIFSVRYLDMLRLGFKLTWMVLGV~TVTAEVAGKTVLNQGLA
FG09796.1 	~WNNPR1ASAIAYATIVSAIFAARYLDVLRWGLKTSWMVLGV~TILAEVLGKVILNNGLA
AN5404.1  	0WEQPR~ATAASFASVVAFIFAARYLPLLRWFFKFLYLVLGV1TAAAEIGGRVVLSQGLT
          	 *::** *:. :: ::*: **:.*** ::*  :*    **.. *  **: *:.:*. *::

MG04197.1 	SQLRPRRYYTVSRETLESMTGDVHELLNFFCIESQRILFAENVTVSAV0ACVGALISYWL
NCU06361.1	TQLRPRKYYTVSKETLDSAIGDVHELVNFFVIEAQRILFAENVYVSGL~VAIAAFFAYYL
FG09796.1 	TQVRPRRYYTVPRETLDAVIGDVHELINFFVIEAQRIIFAENVAASAA0AFVAAFISYYL
AN5404.1  	SSFRPRKYYTLPRETIEAVLEDFQQLVDFLFIEFQRILFAENAVHTTA0AAAAAFSAYWL
          	:..***:***:.:**:::   *.::*::*: ** ***:****.  :   .  .*: :*:*

MG04197.1 	VKIVPYWGLALMTTTLAFVAPLIYKTNKEMIDEHLKQVSSIVESQTNQLKEVAGKHTAQA
NCU06361.1	VKIVPYWGLALIATTLAFLGPLVYKTNKEFIDAQLHRASEIIGAQTAQLKTAAQQNSEKA
FG09796.1 	VKLVPVWGLTLIGTTVAFVVPLIYTSNQELIDEQLHHASELINNQTAQIQSVASKQMEQV
AN5404.1  	IKILPFWGLSLLAVTIAYLGPFVYLNNQEIIDAQIENVRQMVGSQANQLKDLAEERTYHA
          	:*::* ***:*: .*:*:: *::* .*:*:** ::... .::  *: *::  * :.  :.

MG04197.1 	TELTKQYMGDCTAKAQSLLRGRSP-------------ETVSKTPAAPAP-ADN--LAHDL
NCU06361.1	TQLTKQFVGEYTAKAQTLVSKATP-------------ASLHGEEHTNGHANKSELRDADF
FG09796.1 	SNISKQYVGDYSGKVQDLLQGKTPSRQKIDMPQNSNSALPAKQPQFPSPPTDEPIASANP
AN5404.1  	TGVVKQYVGDYSNKAQEYIGRRSASP-------EVAKGPAAGPVVKREPEPEAVVKTSDF
          	: : **::*: : *.*  :   :.:        .  .              .      : 

MG04197.1 	PSAPT-----------TDIKTTEP------ITEEKEPLLAL
NCU06361.1	PPAPQ-----------ADIQ-TEPVVPVTVKSGEQQPLLI-
FG09796.1 	PQIPTPAALREEINEPTAIGTAAPEVPLENVVPAKEPMLAS
AN5404.1  	PEAPK----------------VEPVAQSIETQSEKEPLLAI
          	*  *                 . *          ::*:*
```
